# Brazil’s sugarcane embitters the EU-Mercosur trade talks

**DOI:** 10.1038/s41598-021-93349-8

**Published:** 2021-07-02

**Authors:** Marco Follador, Britaldo Silveira Soares-Filho, George Philippidis, Juliana Leroy Davis, Amanda Ribeiro de Oliveira, Raoni Rajão

**Affiliations:** 1 Joint Research Centre, Bio-Economy Unit, European Commission, Ispra, Italy; 2 Centre for Remote Sensing (CSR), Federal University of Minas Gerais, Belo Horizonte, Brazil; 3 Aragonese Agency for Research and Development (ARAID), Centre for Agro-Food Research and, Technology (CITA), Agrifood Institute of Aragón (IA2), Government of Aragón, Saragossa, Spain; 4 Joint Research Centre, Economics of Agriculture Unit, European Commission, Seville, Spain; 5 Laboratory of Environmental Services Management (LAGESA), Federal University of Minas Gerais, Belo Horizonte, Brazil

**Keywords:** Environmental impact, Climate-change mitigation, Projection and prediction

## Abstract

The Brazilian government’s decision to open the Amazon biome to sugarcane expansion reignited EU concerns regarding the sustainability of Brazil’s sugar sector, hindering the ratification of the EU-Mercosur trade agreement. Meanwhile, in the EU, certain conventional biofuels face stricter controls, whilst uncertainty surrounding the commercialisation of more sustainable advanced-biofuels renders bioethanol as a short- to medium-term fix. This paper examines Brazil’s land-use changes and associated greenhouse gas emissions arising from an EU driven ethanol import policy and projections for other 13 biocommodities. Results suggest that Brazil’s sugarcane could satisfy growing ethanol demand and comply with EU environmental criteria, since almost all sugarcane expansion is expected to occur on long-established pasturelands in the South and Midwest. However, expansion of sugarcane is also driven by competition for viable lands with other relevant commodities, mainly soy and beef. As a result, deforestation trends in the Amazon and Cerrado biomes linked to soy and beef production could jeopardize Brazil’s contribution to the Paris agreement with an additional 1 ± 0.3 billion CO_2_eq tonnes above its First NDC target by 2030. Trade talks with a narrow focus on a single commodity could thus risk unsustainable outcomes, calling for systemic sustainability benchmarks, should the deal be ratified.

## Introduction

European Union (EU) efforts to maintain the safe operating space of our planet include (inter alia) the transformative capacity of its bioeconomy—a highly diverse collection of activities covering food, feed, industry, and energy applications. The EU bioeconomy strategy^1^ is an ambitious initiative to convert biodegradable renewable sources of biomass into desirable market (i.e., value added and employment) and non-market (i.e., ecosystems services, carbon sinks) outputs, with a view to establishing a sustainable twenty-first century model of development. To ensure sufficient biomass availability to meet this goal, the EU will inevitably require reliable and sustained access to third country supplies, as a means to bypass the limits of domestic biomass production that must comply with its environmental and climate targets^2^. In this context, tropical agriculture could play an important role in promoting the EU’s energy transition away from fossil fuels and towards biofuels, given its ability to grow crops that are highly energy efficient. Of particular note, Brazil’s first-generation ethanol avoids between 69–89% of CO_2_e emissions in comparison with regular fossil fuels, while sugar-beet ethanol avoids only between 35–56% and corn-based ethanol emits up to 38% more than regular fossil fuels^3,4^. International trade agreements have therefore emerged as a potential policy platform to guarantee the stability of EU supply chains. However, the EU’s growing demand for biofuels may lead to spillovers^5^, such as increasing deforestation due to feedstock production together with high greenhouse gas (GHG) emissions that could jeopardise the climate benefits stemming from the energy transition^6^. A priority of the EU Green Deal^7^—the new action plan to make the EU’s economy sustainable—is thus to consider the implications of imports, while establishing trade regulations that may mitigate the risks related to the imports of biofuels^8^.

The EU–Mercosur trade deal, agreed in principle on June 28th 2019, promotes efforts to pave a common road towards sustainable growth across its global value chains through a green alliance with its trading partners, though it still lacks a clear mechanism to trace and monitor commodity origin and production^2,9^. The deal is to grant preferential EU market access to South American bio-commodities through tariff rate quotas (TRQs) on sensitive agricultural products, such as sugar and ethanol, conditional on environmental and social standards.

Brazil, the most important member of Mercosur, in terms of its economy and agricultural output, managed to reduce deforestation in the Amazon by 84%, while increasing agricultural production from 2004 to 2012^10^. Zoning laws banning subsidies to sugar and ethanol production and a deforestation moratorium for soy in the Amazon have sent strong signals that Brazil was on the right track to become an important source of sustainable biofuels. However, since 2012 deforestation has been on the rise again with 2020’s rate increased by 140% in relation to that of 2012^11^. In 2019 Brazil unexpectedly revoked the Agro-Ecological Zoning (AEZ) decree for sugarcane that forbade its expansion into the Amazon and other sensitive biomes^12^, thereby reigniting EU concerns regarding the sustainability of Brazil’s sugarcane production. In addition, the controversial dismantling of Brazil’s environmental policies together with the revelation that a large share of EU imports of beef and soy from the country were produced on illegally deforested lands^13^ has complicated the ratification process of the EU-Mercosur trade deal^14^.

Given uncertainties surrounding the potential mass-scale commercialisation of more sustainable advanced generation of biofuels, there is a renewed interest in first generation bioethanol as a short to medium-term fix to achieve EU decarbonisation targets. Nonetheless, public policy support for conventional liquid biofuels has also courted considerable controversy on GHG emissions leakages from direct (LUC) and indirect land-use change (iLUC), as well as feed and food security. To tackle these concerns, the EU launched a series of measures and initiatives. The Fuel Quality Directive laid out a roadmap for a set of credible criteria for the exclusive adoption of sustainable biofuel usage^15^. Subsequently, the EU revised the Renewable Energy Directive (REDII) and devised new environmental criteria for biofuel feedstock, limiting the use of highly iLUC-risk biofuels, specifically due to conversion of native vegetation to croplands^8^. Most recently, under the auspices of the Green Deal, the EU plans to bind its sustainability criteria to its trade relations with external partners. As part of this process the REDII will set the EU-wide renewable energy target to a minimum of 32%, while imposing restrictions on the use of palm-oil-based biofuels. In particular, after identifying that between 2008–2015, 45% of the expansion of palm oil took place in areas of high carbon stocks^16^, the EU is phasing-out biofuels linked to deforestation by 2030. Hence, palm oil biofuels (with some exceptions) will be disqualified as eligible for EU subsidy support and so treated as a regular fossil fuel^17^. As a key player in global bioethanol markets, Brazil could feature as a major replacement supplier. The compliance of Brazilian bioethanol with REDII criteria is therefore the prerequisite for allowing Brazilian producers to take full advantage of the EU-Mercosur trade rate quotas and a significant step forward in the ratification process of the trade agreement. To shed light on this issue, here we examine the future sustainability of EU imports of Brazilian bioethanol by quantitatively assessing the impacts of increased EU demand for bioethanol in terms of its implications for sugarcane expansion and associated land-use change in Brazil. A key issue is whether the direct and indirect land-use changes arising from such a demand increase would comply with EU environmental and sustainability criteria.

Our study assesses a scenario in which to meet its first-generation biofuel mandate, the EU substitutes all biodiesel with bioethanol by 2030. To do so, we employ a state-of-the-art global trade simulation market model with a biobased focus, called MAGNET, to estimate the EU import demand for Brazilian bioethanol. The import demand trends from MAGNET are then inputted into a spatially-explicit land-use model of Brazil (Otimizagro) to forecast the resulting land-use changes. The use of a national model at a high spatial resolution (6.25 ha) is key to properly represent the diversity and complexity of Brazil’s territory, including climates, socioeconomic conditions and regional governance systems. With a coverage of fourteen main crops, Otimizagro^18^ simulates detailed land-use spatial patterns resulting from the expansion of sugarcane and other crops, forest plantation, secondary vegetation regrowth and deforestation trends together with resultant GHG emissions, thus reducing uncertainties surrounding potential LUC and iLUC^19^ in Brazil.

## Results

### EU demand for Brazilian ethanol by 2030

The MAGNET model is used to simulate by 2030 the assumed phasing out of EU biodiesel production (POB-Phase Out of Biodiesel scenario) with compensating rises in its bioethanol capacity in order to hit first-generation biofuel mandate targets (Supplementary Table S1). The POB scenario is built directly upon the bioeconomy-baseline in MAGNET, as described in the Supplementary Information (Supplementary Information S1). The main model drivers behind this medium-term scenario are worldwide country projections of economic growth and population, biophysical (land productivities) and energy related drivers (fossil fuel prices, energy consumption and production trends) and the progressive expected implementation of EU first-and advanced-generation biofuel mandates^20,21^. In accordance with this scenario, EU imports of ethanol rise rapidly after 2020, leading to a larger EU reliance on imports from Brazil^22^. By 2030, the EU share of Brazilian bioethanol exports is expected to be 30% (1.13 billion litres), well above the 0.18 billion litres projected from a baseline scenario (Supplementary Figure S1). Total POB ethanol production reaches 52.24 billion litres in 2030. From a trade policy perspective, EU bioethanol imports rise above the TRQ limit set by the EU-Mercosur deal (650 thousand tonnes) in 2027. The Brazilian production of sugar achieves 52.1 million tonnes in 2030^23^. The total area of sugarcane to meet the demand for ethanol and sugar is 14.8 million hectares (Supplementary Figure S2), hence an increase of 45% (4.6 million hectares). Our figures, derived from projections of sugarcane productivity from the Brazilian Ministry of Agriculture^23^ and ethanol/sugar conversion factors from the National Company of Supplying^24–31^ (Section S2.1), differ by about 6 billion litres from the recently updated official calculations of the Brazilian Ministry of Mines and Energy (46 billion litres^32^) for a scenario of intermediate growth of sugar-energy sector, due to the implementation of the new national biofuel policy, namely the RenovaBio programme^32^.

### Countrywide land-use changes and sugarcane expansion

**Figure 1:**
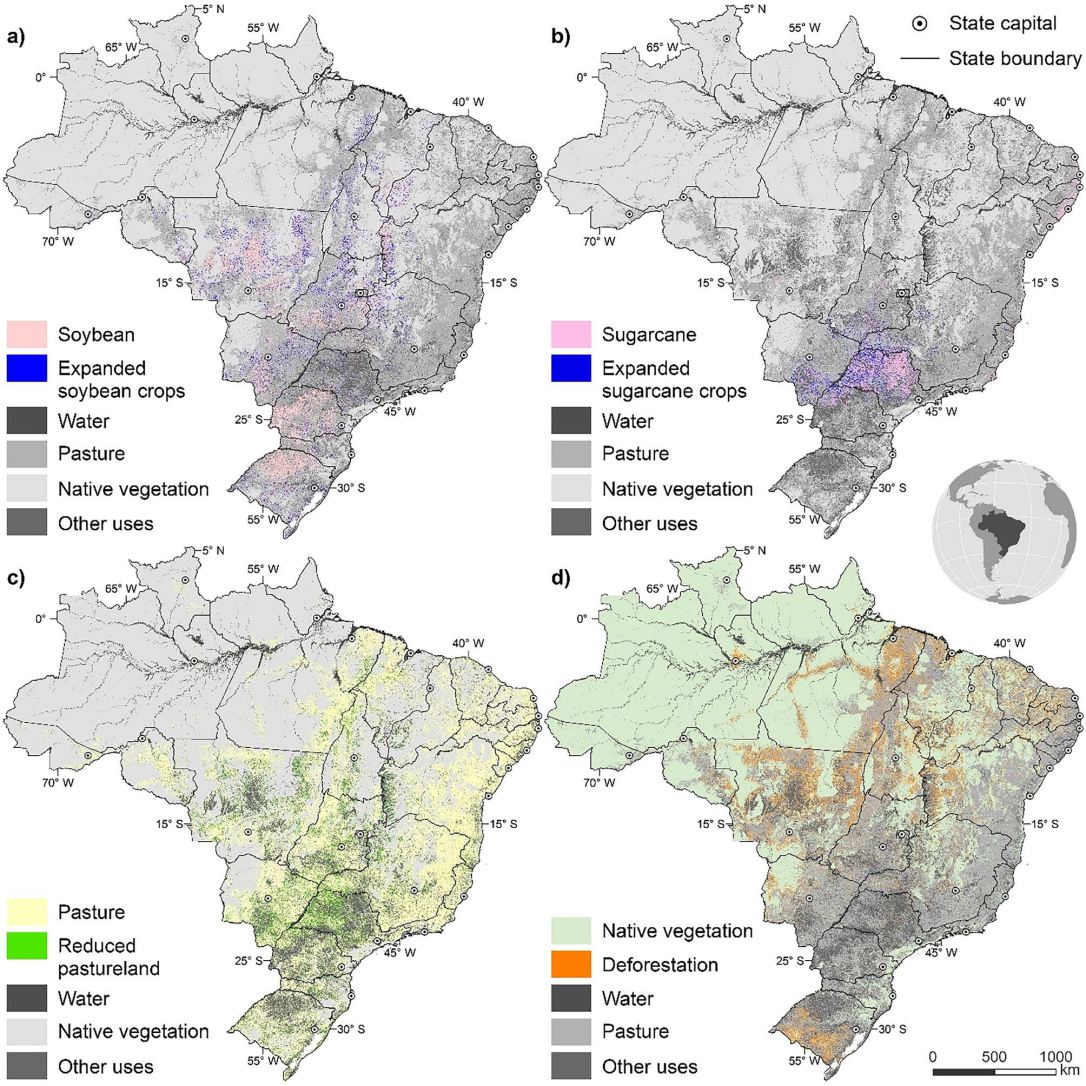
Major land-use transitions. (**a**) Large-scale soybean expansion will take place in the Midwest and Southern Amazon. (**b**) Sugarcane will continue expanding mainly onto pasturelands in the southeast. (**c**) Long-established pasturelands in the Midwest will shrink due to crop expansion. (**d**) Deforestation will advance toward northern regions. Map created using Dinamica EGO 5 (https://dinamicaego.com/).

Land-use changes due to sugarcane production are also driven by competition with other commodities for viable agricultural lands. Therefore, the allocation of sugarcane areas takes place simultaneously with the expansion (or reduction) of the other croplands, forest plantation along with the forest restoration needed to attain the compliance to the Forest Code, the principal law regulating forest conservation on private properties^33^. As a result, Otimizagro fully represents direct and indirect land use changes due to sugarcane expansion, including the displacement of marginal farming and ranching systems in favour of more lucrative crops (Fig. 1).

The projections to 2030 for the main crops (Supplementary Table S2) follow the official estimates of the Brazilian Ministry of Agriculture^23^. Soybean production rises rapidly from 114 million tonnes in 2019 to 163 million tonnes by 2030, with exports representing more than 60% of total production. Double cropping systems that combine first-crop soybeans and second-crop corn account for total corn expansion, with a gradual reduction of first-crop corn. Soybean, sugarcane and second-crop corn areas, which represented more than 60% of the country’s total cropland in 2019, are responsible for the largest increments by 2030—i.e., 34% (12 million hectares), 44% (4.5 million hectares) and 62% (8 million hectares), respectively. Wood consumption from plantations increases from 192 million cubic meters in 2012 to 256 million cubic meters by 2030. As a result, forest plantations expand at a commensurate rate of about 138 thousand hectares (ha) per year in the period 2019–2025, and 159 thousand ha afterwards, reaching 9 million ha by 2030^34^. Deforestation rate trajectories have been derived from an intermediate environmental governance scenario^10^ (Supplementary Information S2.3 and Supplementary Figure S3), which considers a growing political support for predatory agriculture practices, land-grabbing and a progressive dismantling of the country environmental legislation including the Forest Code. This scenario follows closely the rising deforestation trend since 2012 (Supplementary Figure S4). Nevertheless, we also compare GHG emissions from the former scenario with those from a worst-case governance scenario that models the full reversal of the past environmental achievements in Brazil^10^. Regarding forest restoration, we included the targets of the National Plan for Native Vegetation Recovery, which aims at 12.5 million ha of forest restoration by 2035^34^. As a result, there is a gradual increase of secondary forests from 2.6 million hectares in 2019 to 7 million hectares in 2030.

**Figure 2:**
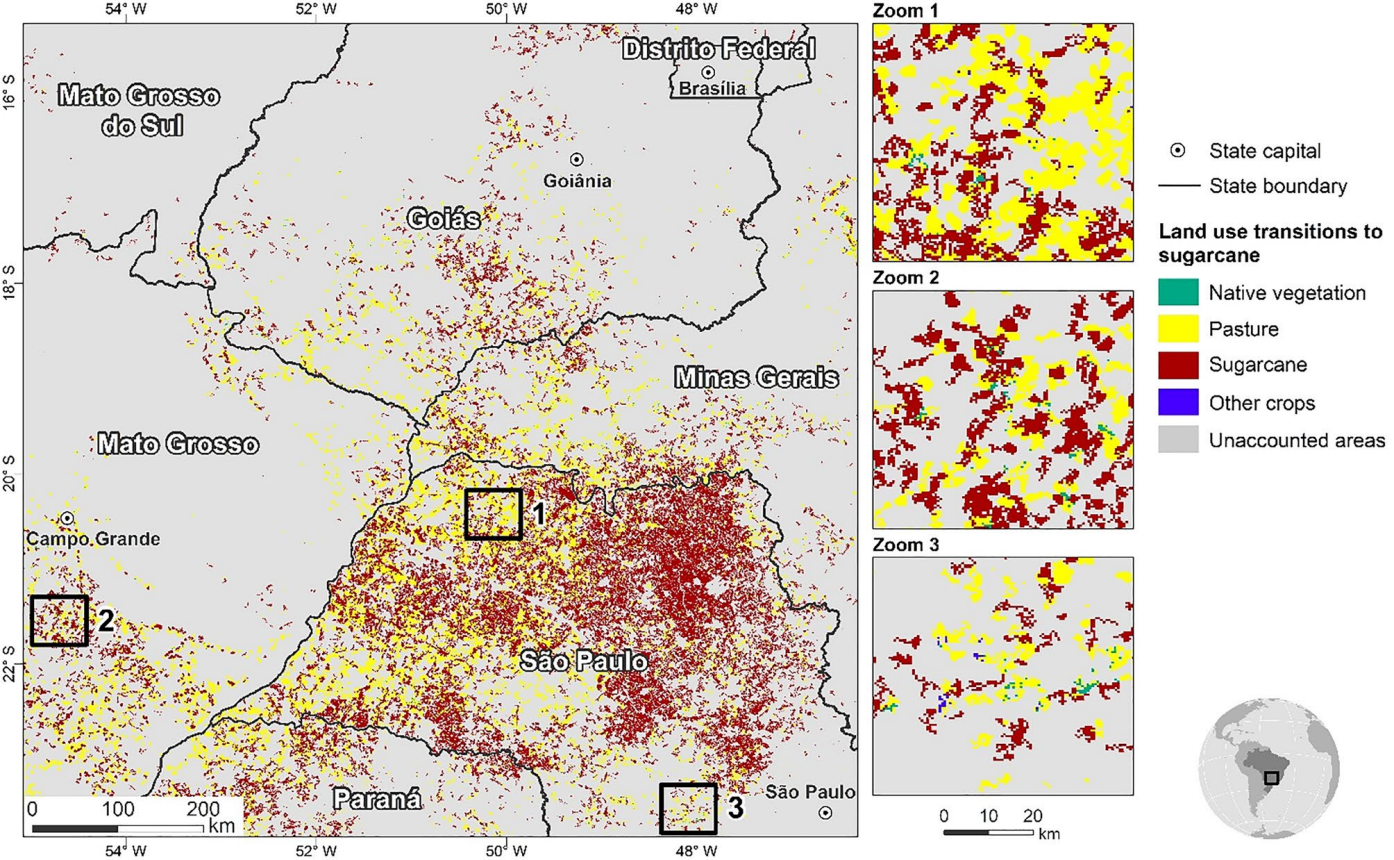
Land use transitions to sugarcane from 2019 to 2030. The vast majority of sugarcane expansion occurs onto pasturelands in the Southeast and Midwest of Brazil. Map created using Dinamica EGO 5 (https://dinamicaego.com/).

Land-use conversions to new sugarcane areas from 2019 to 2030 mainly occur in the Southeast and Midwest of the country (Fig. 2). The largest sugarcane expansion in absolute terms is expected to occur in the State of Sao Paulo (2 million ha), followed by the states of Mato Grosso do Sul (0.9 million ha) and Minas Gerais (0.7 million ha). Mato Grosso do Sul (115%), Minas Gerais (80%) and Goiás (63.5%) are also responsible for the highest rates of increase (Supplementary Table S3).

Most of the sugarcane croplands in 2019 continue to be productive in 2030, representing 69% of the total sugarcane area (Supplementary Table S4). The conversion of native vegetation and other croplands (including food crops) to sugarcane is limited to less than 1% of the total area, resulting in a small loss of forested lands and displacement of other crops. Sugarcane expansion onto pasture accounts for more than 30% of the cumulative expansion of the country’s agriculture from 2019 to 2030 (Supplementary Table S5).

**Figure 3:**
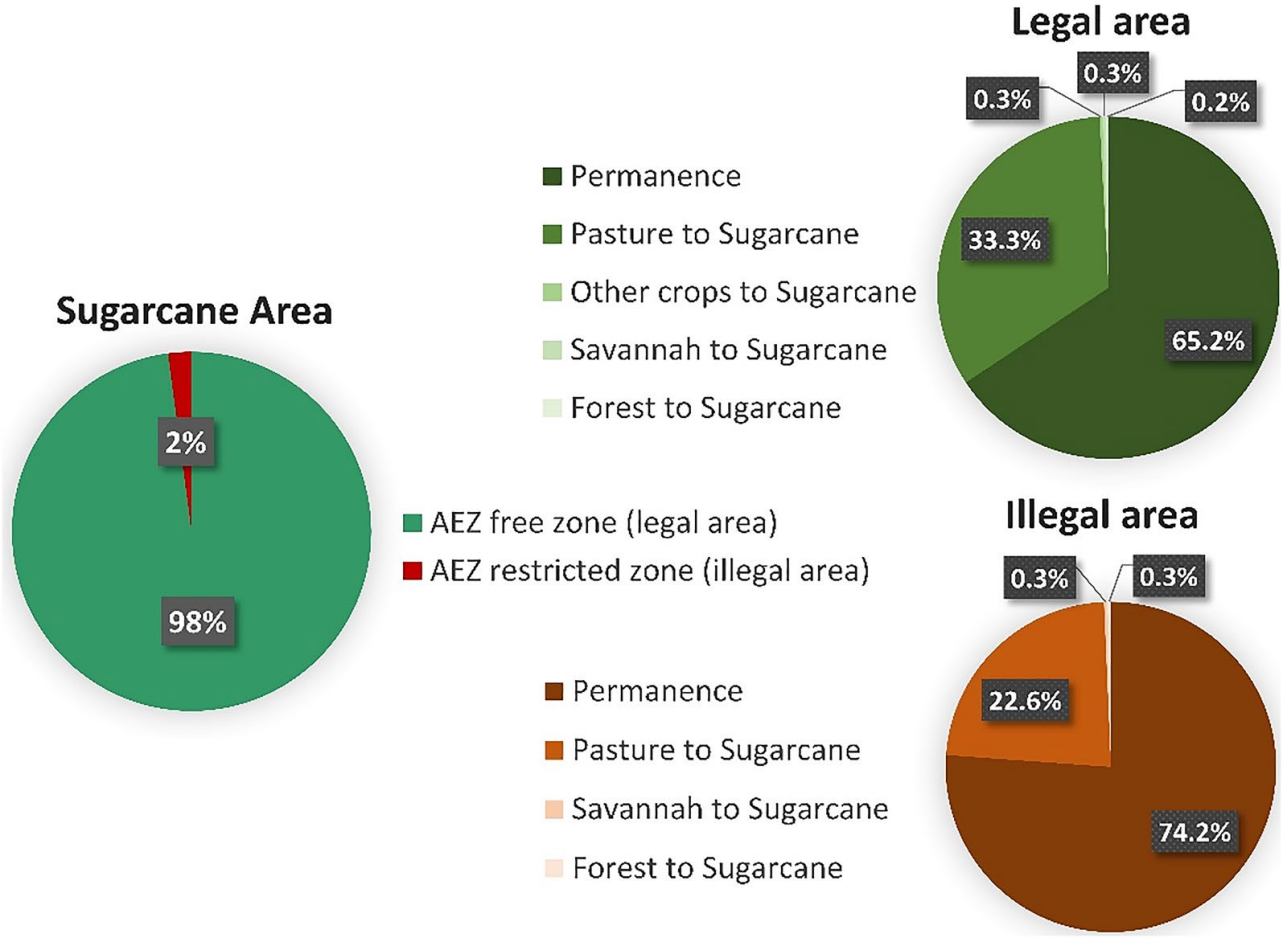
Sugarcane area compliance with the AEZ limits by 2030. Permanence and expansion from 2019 to 2030. Only 2% of the sugarcane area does not comply with the AEZ restrictions (illegal area). Most of this area was already sugarcane in 2019.

The decision of the Brazilian government to revoke the sugarcane zoning decree does not appear to influence sugarcane expansion into the Amazon. Indeed, the results show that only 2% of the total sugarcane area in 2030 (307 thousand ha) is within the AEZ restricted zone, most of which was already sugarcane in 2019 (74%). Similarly, new sugarcane croplands from forest clearance are marginal (Fig. 3).

### GHG emissions from land use, land-use change and forestry (LULUCF)

Roughly 75% of current agricultural land remains so in 2030. The need for new cropland (14 million hectares) expands mainly onto current pastureland (91% of expansion), whilst only 5% and 4% comes from conversion of forest and savannah, respectively (Supplementary Table S5). Clearance of forests and savannahs (39 million ha) is largely linked to land speculation via predatory land-grabbing with subsequent cattle ranching occupation^35^. New land conversion to soybean mainly takes place in the Midwest and northern states (Fig. 1a), with about 7% of total expansion into high carbon forested lands with resultant high GHG emissions, hence a potential threat to the Amazon forest and Cerrado native vegetation^13^. Yet, the extent to which deforestation is due to pasture displacement as a result of large-scale expansion of soybean remains uncertain given the complexity of iLUC domino effects^19^. Accounting for all land-use changes, the country’s annual net LULUCF emission balance rises steeply between 2019 and 2030, from 428 ± 172 to 921 ± 293 million CO_2_eq tonnes (Supplementary Table S6). The difference between LULUCF emissions by 2030 stipulated by Brazil’s First Nationally Determined Contribution (NDC)^36^ (− 131 million CO_2_eq tonnes)^37^ and our results is an additional 1 ± 0.3 billion tonnes. But this gap could be even larger, reaching 1.7 ± 0.4 billion tonnes, if the environmental governance in Brazil further wans (Supplementary Table S6).

**Figure 4:**
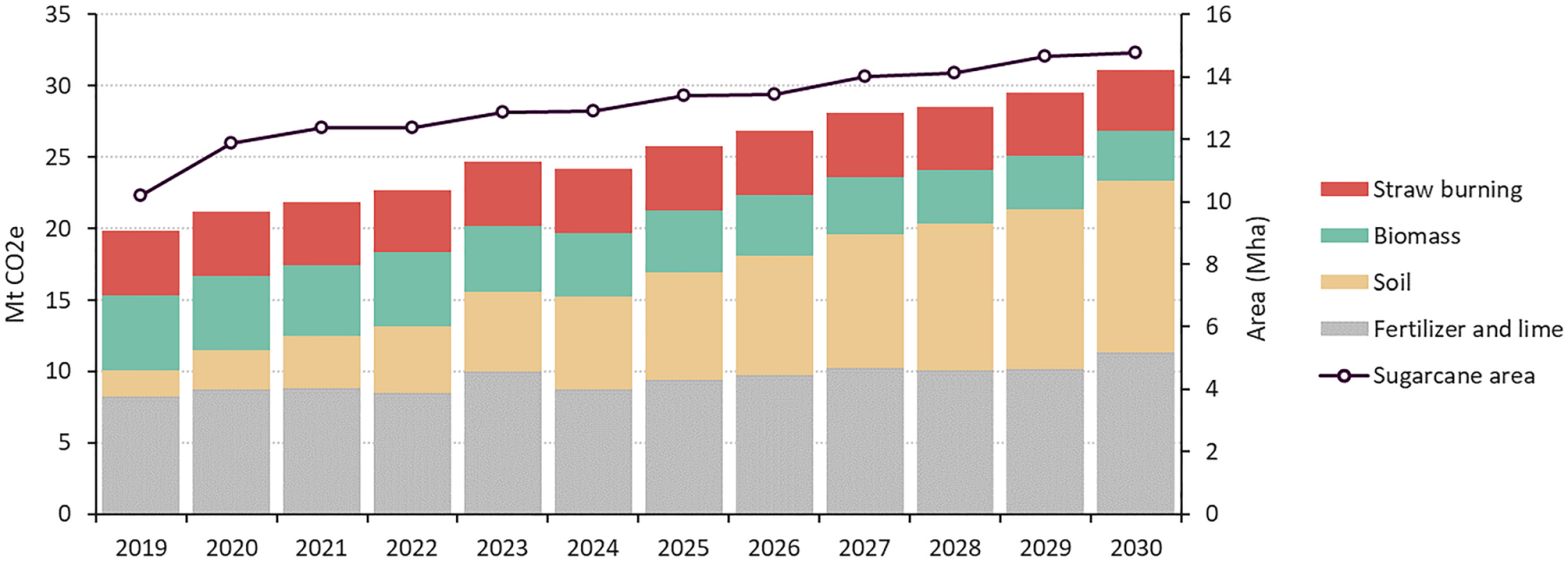
GHG emissions from sugarcane production. LULUCF emissions (from biomass and soil carbon stock changes) represent about 50% of the total, whilst the remaining emissions are due to agricultural practices (fertilizer and lime application and straw burning).

With most sugarcane cropland expansion expected to occur on long-established pasturelands, the GHG emissions from land-use change are limited (48% of total emissions), due to the pasture low carbon content^38^. However, cultivating degraded pasture requires the use of fertilizer (90 kg N/ha, on average) and limestone (2 tonne/ha) to achieve the expected sugarcane productivity per hectare^34^, representing an additional source of GHG emissions (46% of total emissions). In addition, some regions of Brazil, notably the northern states, rely on burning sugarcane straw to facilitate manual harvesting (5% of total emissions). Even though the AEZ envisaged to moderate this practice, only the state of Sao Paulo enacted a law in 2002 that aims to completely phase out the burning of sugarcane straw by 2021. Figure 4 and Supplementary Table S7 show the total sugarcane area and associated GHG emissions from 2019 to 2030. On average, the GHG emission is ca. 1.7 ± 0.17 tonnes of CO_2_eq ha^−1^ year^−1^ (total 24.7 ± 2.3 Mt CO_2_eq year^−1^).

## Discussion

Our study shows that sugarcane croplands could meet domestic and international bioethanol demand without further deforestation. Indeed, most sugarcane expansion would occur at the expense of pasturelands in the Southeast and Midwest regions, given the concentration of sugar and ethanol mills, especially in Sao Paulo State, together with the well-developed system for transportation of ethanol, thereby reduced transportation and production costs. In addition, in this region ranching is, in general, economically less competitive than sugarcane. Converting pasture to sugarcane and achieving commercially viable yields require a substantial application of lime and fertilizers, which represents about 50% of GHG emission from sugarcane cultivation. However, these emissions are far lower than those from deforestation linked to crop expansion. Consequently, this strategy could represent an opportunity for the Brazilian sugarcane industry to meet the rising demand for ethanol and sugar while achieving the country’s sectoral mitigation objectives (i.e., the NDC and Low Carbon Agriculture^39^ targets) along with the compliance with national and international environmental standards (i.e., REDII and RenovaBio environmental criteria). To this end, taking a long-term perspective, the amendment of more sustainable supplies, such as biochar, could further increase soil proprieties and agricultural productivity of degraded pasturelands, while contributing to lower emissions^40,41^. The potential conversion of the Amazon and Cerrado native vegetation to sugarcane could be marginal, resulting in limited LULUCF emissions. Indirect land use changes are also far from certain, since cattle ranching intensification has been the most cost-effective solution to yield land for sugarcane expansion in the southeast of Brazil, especially in regions with easy access to grain production^42–44^.

Even though revoking the AEZ for sugarcane meant a further step toward the weakening of environmental governance in Brazil, its consequence could manifest itself more in terms of tarnishing the image of Brazilian ethanol rather than resulting in a real expansion of sugarcane crops into the Amazon and Cerrado native vegetation. The 2018/2019 sugarcane production in the Amazonian states was less than 1% of country production^45^, and in the absence of restrictions, the sugarcane area is likely to double within the biome by 2030. Nevertheless, more than 97% of production is poised to occur in the mid- and southern Cerrado and Atlantic Forest biomes, with potentially little direct conversion from forests and savannah. This trend is expected to continue in the near future, since most of the projects for new ethanol plants are located near road infrastructure in the southern regions^32^. Moreover, the RenovaBio programme already incorporates sustainability criteria to avoid the use of biofuels grown on lands deforested after December 2017. Together with the Forest Code, these measures—if properly enforced—represent an effective legal tool to ensure that the ethanol supply chain remains deforestation-free. The displacement of other crops could be negligible, thereby avoiding potential concerns about regional food security and market stability.

On the other hand, the country’s agricultural expansion as a whole, raises sustainability concerns. If Brazil’s need for new cropland (14 million ha) could be met solely on existing pastureland (91%), the main driver of deforestation continues to be significant losses of native forest and savannah vegetation (39 million ha) due to land speculation via predatory land-grabbing, with subsequent cattle ranching occupation^46^. The extent to which this is due to pasture displacement as a result of large-scale expansion of soybean onto already cleared areas remains uncertain given the complexity of iLUC domino effects^19^. However, there exists evidence that a share of agricultural commodities employing illegally deforested land is exported from Brazil to the EU market^13^. All of this not only tarnishes the reputation of Brazil’s agribusiness, it also places an additional burden on other countries to mitigate climate change, if Brazil ultimately fails to fulfil its NDC contribution to the Paris agreement^10^.

## Conclusion

Access to the EU single market for third country commodities is subjected to compliance with the EU sustainability criteria. However, to date, only the imports of a few commodities have been clearly regulated, for which compliance can be assessed. Among them, biofuels must comply with the environmental standards set by the updated EU Renewable Energy Directive (REDII), which limits biofuel feedstock expansion onto lands with high carbon stocks. The decision of the Brazilian government to open the Amazon and Pantanal biomes to sugarcane plantations thus reignited EU concerns about the sustainability of Brazilian ethanol—which has long been a sticking point in the 20 years’ trade negotiations—complicating the ratification of the EU-Mercosur deal. Our study shows that the Brazilian sugarcane sector could meet the soaring domestic and international demand for ethanol without further deforestation. Nevertheless, this will require proper agricultural practices along with a sustainable intensification of ranching to free up land for agricultural expansion and as a result avoid iLUC in the form of pasture-displacement into distant forest areas.

Although the increase of Brazilian ethanol production would still comply with the REDII environmental criteria, the recent high deforestation rates in the Amazon and Cerrado biomes could further undermine the ratification of the EU-Mercosur trade deal. The difference between the country’s First NDC stipulated LULUCF emissions by 2030 and our results is an additional 1 ± 0.3 billion CO_2_eq tonnes, placing Brazil’s contribution to the Paris Agreement at risk. Deforestation linked to production of other commodities exported to the EU, such as soybeans or meat, are not regulated by clear EU sustainability criteria, leading to potential disputes between the parties. Trade policy based on narrow attention to single commodities therefore risks unsustainable outcomes and could aim at the wrong target. The EU should negotiate responsive international agreements based on enforceable environmental criteria for all traded key commodities within a systemic, science-based understanding to halt EU-driven deforestation^47^ and meet the Green Deal objectives of promoting sustainability across the whole supply chain, whose effectiveness has been recently questioned^2^. This must be bolstered, in parallel, by diplomatic efforts to support socioeconomic growth built upon Brazil’s past history of strong environmental achievements^48^.

## Methods

### Modelling framework

Global Computable General Equilibrium (CGE) models have emerged as a tool for international impact assessment. Due to their considerable geographical coverage and trade connected macroeconomic systems, they are suitable for assessing the synergies and trade-offs, both domestic and internationally, arising from public policy. In this context, various CGE studies have examined the impacts of direct and indirect land-use change due to biofuel policies^49,50^. On the other hand, the lack of a fine spatial resolution in multi-region CGE modelling justifies a soft-coupling with a spatially-explicit land-use model. A high spatial resolution of land-use models allows the inclusion of detailed geographic features, such as terrain, soils, land tenure, land use zoning and other features, in order to provide a realistic picture of land use trends across the country as well as associated GHG emissions. Thus, we established a comprehensive methodological procedure by loosely coupling the global economic market simulation model, MAGNET, and the spatially explicit land-use model Otimizagro. This allows moving from a regional outlook on socioeconomic trends to a subnational analysis of land use patterns, with the proper spatial resolution (6.25 ha) to assess the compliance with EU environmental criteria for biofuels production.

### MAGNET model

MAGNET^51^ is a class of CGE global market simulation model calibrated to an in-house developed biobased derivative of the publicly available Global Trade Analysis Project (GTAP) database^52^. With a base year of 2011 and 141 regions of the world, GTAP version 9 data provides detailed information on the structure of demand at pre- and post-tax prices for 57 activities, and private and public purchases. In addition, the data are complemented with gross bilateral trade flow data between all regions and trade protection instruments and transport costs. The GTAP data also capture interregional savings and investment flows. An in-house MAGNET variant of the GTAP database extends considerably the coverage of biobased activities, incorporating (*inter alia*) an explicit separation of conventional liquid biofuel activities^21,53^. Consequently, it serves as an ideal platform upon which to analyse changes in EU biofuel policy on third countries.

The accompanying model employs mathematical functional forms to capture the tenets of neoclassical economic theory to motivate the behaviour of agents (firms, consumers, investors). Additional market clearing and accounting equations enforce the underlying ‘equilibrium’ conditions of the model database, namely that supply equals demand in each market, economic profits remain zero and that the value of output, income and expenditure within each macro-economy are balanced. Furthermore, the flow of transactions of goods and services within and across national boundaries is supported by price transmission equations. To ensure a model solution, the number of equations and endogenous variables (typically prices and quantities) in the model system must be equal, known as the model ‘closure’. Remaining variables (i.e., tax rates, technology changes, endowment changes) are held exogenous. Imposing ‘shocks’ to key technology or policy variables, through a closed circular flow of accounting and market clearing equations, the model arrives at a new vector of prices which ensures a new counterfactual equilibrium solution. Further details on model assumptions and limitations in Supplementary Information (Supplementary Information S1 and Supplementary Table S1).

### Otimizagro model

Otimizagro is a nationwide, spatially-explicit model that simulates land use, land-use change, forestry, deforestation, regrowth, and associated GHG emissions under various scenarios of agricultural land demand and deforestation policies for Brazil^10,13,18^. Otimizagro simulates nine annual crops (i.e. soy, sugarcane, corn, cotton, wheat, beans, rice, manioc, and tobacco), including single and double cropping; five perennial crops (i.e. arabica coffee, robusta coffee, oranges, bananas, and cocoa); and plantation forests. The model framework, developed using the Dinamica EGO platform^54^, is structured in four spatial levels: (i) Brazil's biomes, (ii) IBGE micro-regions, (iii) Brazilian municipalities, and (iv) a raster grid with 6.25 ha spatial resolution. Concurrent allocation of crops at raster cell resolution is a function of crop aptitude and profitability, calculated using regional selling prices, production and transportation costs. When the available land in a given micro-region (or other specified spatial unit) is insufficient to meet the specified land allocation, Otimizagro reallocates the distribution of remaining land demands to neighbouring regions, creating a spillover effect. Future demand for crops, and deforestation and regrowth rates are exogenous to the model^10,23,34^ (Supplementary Information S2). The probability of deforestation is a function of spatial determinants, such as distances to roads and previously deforested areas. To account for GHG emissions from land-use, land-use change and forestry (LULUCF), Otimizagro calculates emissions and removals from biomass and soil according to the Third National Communication (TNC) of Brazil to the United Nations Framework Convention on Climate Change^55,56^. TNC database includes a biomass map (Supplementary Figure S5), a reference soil carbon stock map (Supplementary Figure S6) and carbon emission/removal rates (Supplementary Table S8, Supplementary Table S9 and Supplementary Table S10). Biomass parameters include live (aboveground and belowground) and dead carbon pools. In comparison with other biomass maps available for Brazil, the aboveground pool has intermediate average values^57^. For biomass calculation, in the initial year, native vegetation categories assume the values of the biomass map. Regrowth is assumed to stabilize at 44% of the original vegetation biomass. Biomass values are assigned to anthropic land-use categories according to Supplementary Table S9. For carbon soil, the model assumes that the stocks begin in equilibrium; thenceforth, the reference soil carbon stock is multiplied by soil carbon stock change factors. Annual carbon emissions are calculated cell by cell and attributing carbon stock changes according to a set of conditions. Soil carbon stock change follows equation Eq. S1. The stabilization threshold is the IPCC default time of 20 years. The model also calculates emissions from fertilizers, limestone and pre-harvest using the TNC emission factors (Supplementary Information S5.2, Supplementary Information S5.3 and Supplementary Information S5.4). Our estimates of GHG emissions include uncertainty thresholds from sensitivity analyses and biomass field measures^58^ (Supplementary Information S4).

## Supplementary Information

Supplementary Information.

## Data Availability

Model input and output maps available at maps.csr.ufmg.br.
