# Supplementary material for: Brazil’s sugarcane embitters the EU-Mercosur trade talks
Source: Sci Rep. 2021 Jul 2;11:13768. doi: 10.1038/s41598-021-93349-8 (PMC8253810; doi:10.1038/s41598-021-93349-8)
Supplement: Supplementary file 1 — Supplementary Information. [file 41598_2021_93349_MOESM1_ESM.docx]

Online Supplementary Information

# Brazil’s sugarcane embitters the EU-Mercosur trade talks

# Marco Follador¹*, Britaldo Silveira Soares-Filho^2^, George Philippidis^3,4^, Juliana Leroy Davis^2^, Amanda Ribeiro de Oliveira^2^, Raoni Rajão^5,2^

# Affiliations

# ¹European Commission, Joint Research Centre, Bio-Economy Unit, Ispra, Italy.

# ^2^Centre for Remote Sensing (CSR), Federal University of Minas Gerais, Belo Horizonte, Brazil.

# ^3^Aragonese Agency for Research and Development (ARAID), Centre for Agro-Food Research and, Technology (CITA), Agrifood Institute of Aragón (IA2), Government of Aragón, Zaragoza,

# ^4^European Commission, Joint Research Centre, Economics of Agriculture Unit, Seville, Spain.

# ^5^Laboratory of Environmental Services management (LAGESA), Federal University of Minas Gerais, Belo Horizonte, Brazil.

*Corresponding Author: marco.follador@ec.europa.eu

**S1. Magnet’s socioeconomic and policy scenarios to 2030**

Baseline

Within MAGNET, a bioeconomy-baseline was developed and peer reviewed in-house in unit D.4. of the Joint Research Centre (JRC) of the European Commission. A full description of this baseline can be found in Philippidis et al.^1^. To further accommodate the focus of this study, some additional calibration was added (see discussion below).

The construction of the baseline, or business as usual, scenario for the MAGNET model is conducted over three time periods from 2011 to 2030 (2011–2015; 2015–2020; 2020–2030). Employing secondary data and assumptions, the main market drivers over this period are macroeconomic (real GDP, population), biophysical (land productivities) and energy related (fossil fuel prices, energy consumption and production trends). To further refine the developments on biobased markets and the resulting impacts on third country trade trends, additional exogenous impacts are introduced relating to environmental- (worldwide GHG reductions), EU agricultural support, bioenergy and trade policies. A detailed discussion of all these assumptions and modelling approach is available online in Philippidis et al.^1^. In the context of the current research, the key baseline assumptions regarding the biofuel market trends are discussed briefly here.

Following Banse et al.^2^, fiscal-neutral first- and second-generation biofuel mandates consistent with the recent EU energy package finalized in June 2018, are assumed. Thus, in the baseline, the EU-wide average first-generation biofuel mandate reaches 7% by 2020 and is maintained to 2030. Taking a time-linear approach, advanced biofuel blending mandates of close to zero in the benchmark year (2011) to 3.5% in 2030, are implemented.

Given the specific focus on EU-Brazil trade relations, additional shocks to the baseline description in Philippidis et al.^1^ have been introduced. In seeking to reinforce the sustainability criterion underlying the EU’s first-generation biofuels policy, there was ongoing debate within the European Parliament to phase out palm-oil based biodiesels^3^, which was eventually formalized within the June 2018 agreement with a complete withdrawal of palm-oil usage in conventional biofuels by 2030. Thus, all EU imports of palm oil from Asia are reduced to zero. Employing data from the Brazilian Ministry of Mines and Energy^4^, additional Brazilian bioethanol export trade share shocks (principally to the EU, USA, South Korea and Japan) have been introduced to mimic the export quantity trends from official Brazilian government sources and projections. To reflect their key status as determinants of marginal changes in land usage in Brazil, further baseline shocks have been imposed on historical and projected production trends for Brazilian bioethanol (billions of litres) based on data from EPE^4^. Further examination of the modified baseline, revealed that the outcomes for Brazilian oilseeds (i.e., soybean) and sugar tracked reasonably well the production and export trends reported by official Brazilian sources^5^.

On top of this baseline, a phasing-out of biodiesel scenario (POB) is constructed which deviates from the baseline over the decade of 2020–2030. Assuming the same conventional biofuel mandates as in the baseline, the EU’s first-generation (or conventional) biofuels mandate is now solely met by bioethanol. As a result, biodiesel, its principal feedstocks (oilseeds, crude vegetable oil) and its animal feed by-product (oilcake), are expected to take a hit. Similarly, greater EU dependence on bioethanol requires increased imports from third country sources, in particular Brazil. The resulting impacts on EU bioethanol imports from Brazil and Brazilian bioethanol production, are presented in the results section.

A limitation in the MAGNET modelling exercise is the impact of the trade elasticities employed in the baseline. Here, it was decided to keep the same “Armington” or trade elasticities as established in our original baseline, although it is true that different trade elasticity magnitudes can provoke deviations in the projections of EU import responses of bioethanol.

**Supplementary Table S1**: assumptions for the construction of the **Phase-out-biodiesel** (POB) scenario in MAGNET

| **Periods** | **Assumptions** |
| --- | --- |
| 2011-2015; 2015-2020; 2020-2030 | Real GDP and population growth projections from European Commission (EC, 2016). |
|  | Land productivity growth: projections from von Lampe et al., (2014). |
|  | Global fossil fuel price projections for coal, crude oil and gas (World Bank, 2017) for each period. |
|  | Greenhouse gas emissions reductions from European Commission (EC, 2016) for each period. |
| 2011-2015 | **Trade Policy (Trade)** |
|  | EU28 Enlargement elimination of tariffs between the EU and Croatia |
|  | Extension to Croatia of an EU common external tariff (CET) on third country trade and reciprocal third country CETs extended to Croatia as an EU28 member. |
|  | **Agricultural Policy** |
|  | Continued phasing in of decoupled payments for 2004 and 2007 accession members |
|  | Targeted removal of specific pillar 1 coupled support payments: Seeds, beef and veal payments (except the suckler cow premium) decoupled by 2012, Protein crops, rice and nuts decoupled by 1 January 2012 |
|  | Re-coupling of support under the article 68 provision |
|  | Greening of 30% of first pillar payments |
|  | Pillar 2 payments to the EU Member States under the financial framework |
|  | Abolition of raw milk (2015) quota |
|  | **EU Biofuels Policy (BF)** |
|  | 1^st^ generation EU average bio-fuel mandate of 5.75% |
| 2015-2020 | **Trade Policy (Trade)** |
|  | EU-Canada trade shocks with HS6 product exceptions tariffs |
|  | EU-Vietnam trade shocks with HS6 product exceptions tariffs |
|  | **Agricultural Policy (CAP)** |
|  | First and second pillar payments follow financial framework budget envelopes. |
|  | Abolition of raw sugar (2017) quotas |
|  | **EU Biofuels Policy (BF)** |
|  | 1^st^ generation bio-fuel mandate of 7% |
|  | Elimination of all palm oil imports flows from Asia to the EU. |
| 2020-2030 | **Agricultural Policy (CAP)** |
|  | 2% p.a. reductions in CAP budget payments. Pillar 1 (coupled/decoupled) and pillar 2 (by rural development measure) payment structures assumed unchanged from 2020. |
|  | **Bio-energy Policy (BF)** |
|  | EU28-wide 1^st^ generation bio-fuel mandate of 7% EU28-wide 2^nd^ generation bio-fuel mandate of 3.5% |

**Supplementary Figure S1:** Total exports quantities from Brazil to the EU, under the baseline and POB scenarios, as modelled by MAGNET.

**S2. Exogenous Input to Otimizagro**

**S2.1. Sugarcane area to 2030**

MAGNET projected the total supply and the EU demand for Brazilian ethanol under two policy scenarios: in the baseline, the ethanol supply rises to 51.6 billion litres, in the phase-out-biodiesel (POB) to 52.2 billion litres. The Brazilian production of sugar has been derived from official projections of the Brazilian Ministry of Agriculture^5^, which estimate a 52 million tons supply in 2030. The total area of sugarcane to meet the demand for ethanol and sugar has been calculated by considering an average Total Recoverable Sugar (ATR) of 129 kg sugar per ton of sugarcane, an 82% effectiveness of milling and productive processes and an increasing land productivity trend in the period from 74 ton/ha to 87 ton/ha^4^. From 2017 to 2030 the sugarcane cropland increases by 45% (4.6 million hectares) in the POB scenario and by 43% (4.4 million hectares) in the baseline.

**Supplementary Figure S2:** Growth of sugarcane area from 2019 to 2030 under the POB scenario.

**S2.2. Crop area projections to 2030**

**Supplementary Table S2**: Projections of the areas (Mha) of the main crops from 2019 to 2030. Source: IBGE^6^, MAPA^5^.

| **Crop** | **Area 2019 (Mha)** | **Area 2030 (Mha)** |
| --- | --- | --- |
| Soybean | 35.9 | 47.1 |
| Corn (2st crop) | 12.9 | 20.9 |
| Corn (1st crop) | 4.89 | 3.28 |
| Bean | 2.77 | 3.19 |
| Wheat | 2.11 | 2.27 |
| Rice | 1.73 | 2.10 |
| Coffee | 1.83 | 1.80 |
| Manioc | 1.21 | 1.19 |
| Feather cotton | 1.63 | 1.16 |
| Cocoa | 0.58 | 0.66 |
| Banana | 0.47 | 0.53 |
| Orange | 0.59 | 0.49 |
| Tobacco | 0.36 | 0.42 |

**S2.3. Deforestation trends to 2030**

The projection to 2030 of deforestation rates for the Amazon and Cerrado biomes is derived from Rochedo et al.^7^ (Figure S2). We selected the intermediate environmental governance scenario (IEG), which assumes the maintenance of current deforestation policies and considers a growing political support for predatory agriculture practices, land-grabbing and a progressive undermining of protected areas legislation and the Forest Code. This scenario follows closely the rising deforestation trend since 2012 (Figure S3). The annual deforestation rates for the other biomes, i.e., Caatinga (4857 km^2^), Pantanal (1010 km^2^), and Pampas (2275 km^2^), are an average from 2002-2010 period from Bustamante et al. (2015)^8^, and for Atlantic Forest (207 km^2^) it was used the average from 2012-2017 period from SOS Mata Atlântica^9^.

**Supplementary Figure S3:** Projections of deforestation rates in the Brazilian biomes from 2017 to 2030. Source: Rochedo et al.^7^ (1000 km^2^ for the Amazon and Cerrado), Bustamante et al.^8^ (average 1000 km^2^/year for the Pantanal, Pampas and Caatinga) and SOS Mata Atlântica^9^ (1000 km^2^/year for the Atlantic forest).

**Supplementary Figure S4:** Amazon historical deforestation (2005-2020), projected scenarios (2018-2030) by Rochedo et al.^7^ and linear trend (2021-2030).

**S3. Land use results**

**S3.1. Expansion of sugarcane sorted by states**

**Supplementary Table S3**: Sugarcane area, expansion (thousand ha) and change (%) from 2019 to 2030 sorted by States

|  | **Sugarcane Area (kha)** | **Sugarcane expansion Area (kha) vs. 2019** | **Sugarcane expansion Change (%) vs. 2019** |
| --- | --- | --- | --- |
| **State** | **2019** | **2030** | **2030** |
| Acre | 3.0 | - | - |
| Alagoas | 411.0 | 0.8 | 0.2 |
| Amapa | 0.2 | - | - |
| Amazonas | 5.7 | - | - |
| Bahia | 121.0 | 32.4 | 26.8 |
| Ceará | 42.6 | 2.8 | 6.5 |
| Distrito Federal | 0.9 | 1.7 | 180.4 |
| Espirito Santo | 78.1 | 13.6 | 17.4 |
| Goiás | 730.0 | 463.0 | 63.5 |
| Maranhão | 49.9 | 0.2 | 0.4 |
| Mato Grosso | 229.0 | 18.5 | 8.1 |
| Mato Grosso do Sul | 778.0 | 891.0 | 115.0 |
| Minas Gerais | 905.0 | 731.0 | 80.8 |
| Pará | 13.1 | - | - |
| Paraíba | 115.0 | 0.3 | 0.2 |
| Paraná | 652.0 | 354.0 | 54.3 |
| Pernanbuco | 342.0 | 4.8 | 1.4 |
| Piauí | 15.8 | 0.3 | 2.1 |
| Rio de Janeiro | 105.0 | 0.5 | 0.4 |
| Rio Grande do Norte | 59.8 | 0.0 | 0.0 |
| Rio Grande do Sul | 37.0 | 1.0 | 2.8 |
| Rondonia | 4.6 | - | - |
| Roraima | 0.6 | - | - |
| Santa Catarina | 12.2 | 0.4 | 3.0 |
| São Paulo | 5,395.0 | 2,051.0 | 38.0 |
| Sergipe | 51.7 | 2.7 | 5.2 |
| Tocantins | 26.2 | - | - |
| **Brazil** | **10,184** | **4,570** | **44.9** |

Note: given the small cropped area, we did not consider the Distrito Federal’s increment as significant to be reported in the manuscript.

**S3.2. Land use transitions to sugarcane**

**Supplementary Table S4**: Land use transitions to sugarcane from 2019 to 2030. Area (Mha) and conversion ratios (%)

| **Transitions 2017-2030** | **Area (Mha)** | **Conversion ratio  (% vs. tot. area)** | **Conversion ratio (% vs. tot. Expansion)** |
| --- | --- | --- | --- |
| Sugarcane permanence | 10.2 | 65.9 | - |
| Pasture to Sugarcane | 4.46 | 33.3 | 97.5 |
| Other crops to Sugarcane | 0.04 | 0.28 | 0.82 |
| Savannah to Sugarcane | 0.05 | 0.37 | 1.07 |
| Forest to Sugarcane | 0.03 | 0.21 | 0.61 |
| Total sugarcane area | 14.8 | 100 | 100 |
| Total expansion area | 4.57 | - | - |

**S3.3. Land use transition matrix**

**Supplementary Table S5**: Land use transition matrix (ha) from 2019 to 2030

|  |  | **2019** | | | | | |  |
| --- | --- | --- | --- | --- | --- | --- | --- | --- |
|  | **Categories of land use** | **Pasture** | **Savannah** | **Forest** | **Regeneration** | **Agricultural area** | **Planted forest** | **Land use 2030** |
| **2030** | **Pasture** | 214,725,275 | 17,506,100 | 19,625,100 | 0 | 2,052,775 | 0 | **253,909,250** |
|  | **Savannah** | 0 | 96,710,625 | 0 | 0 | 0 | 0 | **96,710,625** |
|  | **Forest** | 0 | 0 | 378,365,250 | 0 | 0 | 0 | **378,365,250** |
|  | **Regeneration** | 3,587,450 | 171625 | 423675 | 2,680,800 | 12050 | 0 | **6,875,600** |
|  | **Agricultural area** | 14,725,375 | 627,275 | 744825 | 0 | 59,682,575 | 0 | **75,780,050** |
|  | **Planted forest** | 988,350 | 9,750 | 11,275 | 0 | 29625 | 8,028,850 | **9,067,850** |
|  | **Landuse 2019** | **234,026,450** | **115,025,375** | **399,170,125** | **2,680,800** | **61,777,025** | **8,028,850** | **-** |

**S4. Emissions results**

**S4.1.** **Countrywide LULUCF emissions**

**Supplementary Table S6**: Countrywide LULUCF emission profile (Mtons CO_2_) from 2019 to 2030 under an intermediate (IEG) and weak (WEG) environmental governance scenario

| Million tons CO_2_ | **2019** | **2021** | **2025** | **2030** |
| --- | --- | --- | --- | --- |
| **GROSS EMISSIONS** (IEG) | **798 ± 167** | **894 ± 186** | **1081 ± 227** | **1323 ± 279** |
| **GROSS EMISSIONS** (WEG) | **990 ± 205** | **1289 ± 265** | **1878 ± 387** | **1975 ± 409** |
| - Deforestation (IEG) | 752 ± 150 | 840 ± 168 | 1013 ±203 | 1235 ± 247 |
| - Deforestation (WEG) | 944 ± 189 | 1236 ± 247 | 1810 ± 362 | 1887 ± 377 |
| - Other LUC emissions | 46 ± 16 | 53 ± 18 | 68 ± 25 | 88 ± 32 |
| **REMOVALS** (including CU/IL) | **-368 ± 6.3** | **-374 ± 7.5** | **-387 ± 10.3** | **-402 ± 13.7** |
| - Regeneration | -19 ± 3.8 | -25 ± 5 | -37 ± 7.4 | -52 ± 10.4 |
| - Plantation expansion and other LUC removals | -31 ± 2.5 | -32 ± 2.5 | -32 ± 2.9 | -33 ± 3.3 |
| - Conservation Units/ Indigenous lands (CU/IL) | -318 | -318 | -318 | -318 |
| **NET EMISSIONS** (not including CU/IL) (IEG) | **747 ± 172** | **837 ± 194** | **1012 ± 238** | **1238 ± 293** |
| **NET EMISSIONS** (not including CU/IL) (WEG) | **940 ± 211** | **1232 ± 273** | **1809 ± 397** | **1890 ± 423** |
| **NET EMISSIONS** (including CU/IL) (IEG) | **428 ± 172** | **5196 ± 194** | **694 ± 238** | **921 ± 293** |
| **NET EMISSIONS** (including CU/IL) (WEG) | **622 ± 211** | **915 ± 273** | **1491 ± 397** | **1573 ± 423** |

Uncertainty intervals were calculated considering:

1) Uncertainties related to biomass estimates (Figure S3) and soil carbon stock (Figure S4), which are in the order of 20%^10^;

2) 10% variation in agricultural projections (Table S2)

**S4.2. GHG emissions from sugarcane**

**Supplementary Table S7**: sugarcane GHG emissions from LULUCF and agricultural practices from 2019 to 2030.

|  | **2019** | **2020** | **2021** | **2022** | **2023** | **2024** | **2025** | **2026** | **2027** | **2028** | **2029** | **2030** | **Total** |
| --- | --- | --- | --- | --- | --- | --- | --- | --- | --- | --- | --- | --- | --- |
| **Sugarcane area (Mha)** | 10.1 | 11.8 | 12.3 | 12.3 | 12.8 | 12.9 | 13.4 | 13.4 | 14 | 14.1 | 14.6 | 14.7 | 157 |
| **Sugarcane emission (Mtons CO_2_)** | 16.8 ± 1.4 | 17.8 ± 1.6 | 18.4 ± 1.7 | 19.3 ± 1.9 | 23 ± 2 | 22.7 ± 2.1 | 24.3 ± 2.3 | 25.7 ± 2.5 | 26.9 ± 2.7 | 27.7 ± 2.8 | 28.6 ± 3 | 30.7 ± 3.1 | 296.6 ± 28.6 |
| **- LULUCF** | 7.1 ± 1.4 | 8 ± 1.6 | 8.6 ± 1.7 | 9.8 ± 1.9 | 10.2 ± 2 | 10.9 ± 2.1 | 11.8 ± 2.3 | 12.6 ± 2.5 | 13.3 ± 2.7 | 14 ± 2.8 | 14.9 ± 3 | 15.5 ± 3.1 | 143.1 ± 28.6 |
| **- Fertilizer and lime** | 8.03 | 8.46 | 8.49 | 8.43 | 11.7 | 10.7 | 11.4 | 12 | 12.5 | 12.6 | 12.6 | 14.1 | 138 |
| **- Straw burning** | 1.67 | 1.4 | 1.32 | 1.07 | 1.08 | 1.08 | 1.08 | 1.08 | 1.07 | 1.07 | 1.06 | 1.06 | 15.5 |

**S5. GHG emission estimation**

**S5.1. Details on land use emission estimation (LULUCF)**

**
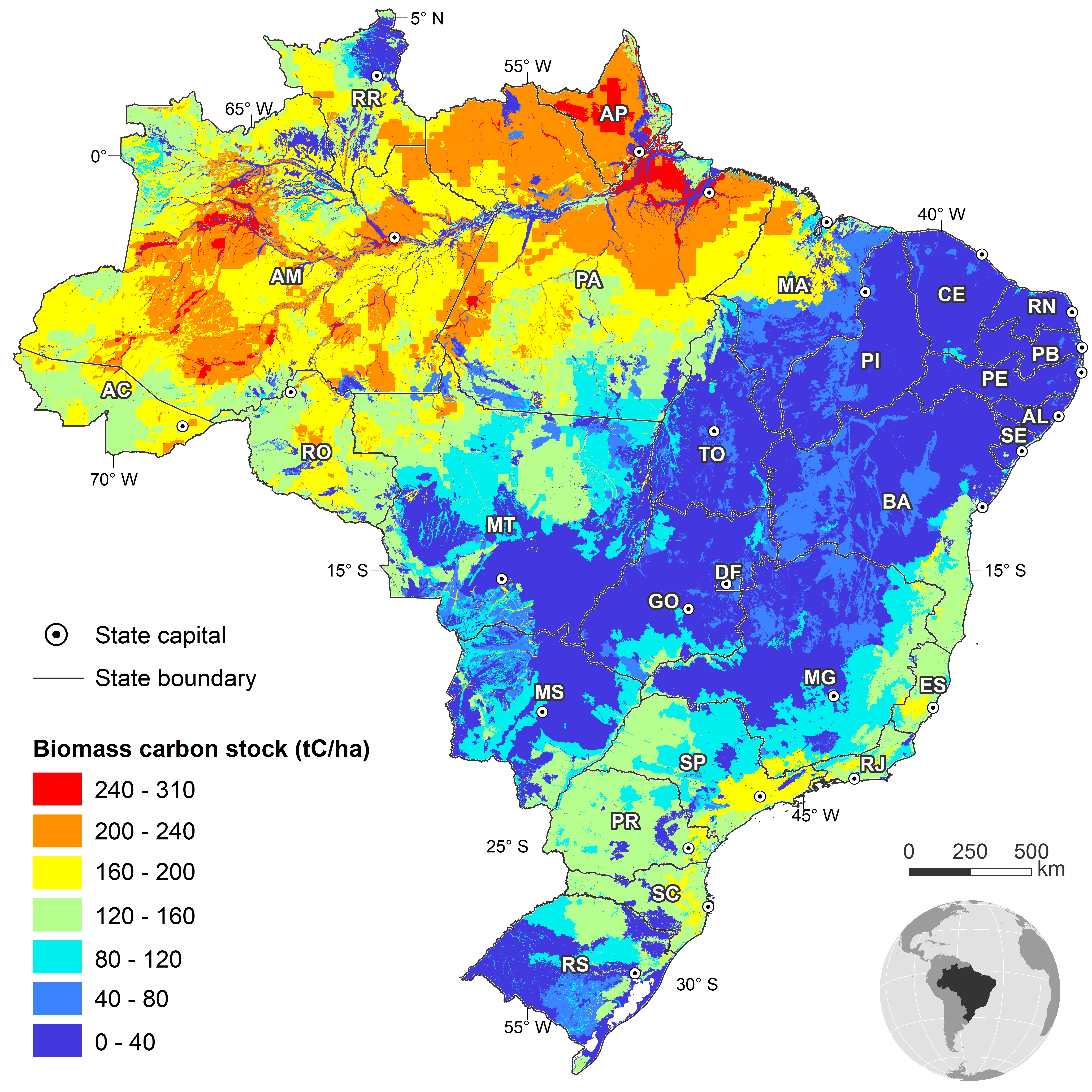
**

**Supplementary Figure S5:** Original vegetational biomass (ton C/ha). Source: Bustamante et al.^8^. Map created using ArcGIS 10.8.1 (www.arcgis.com).

**
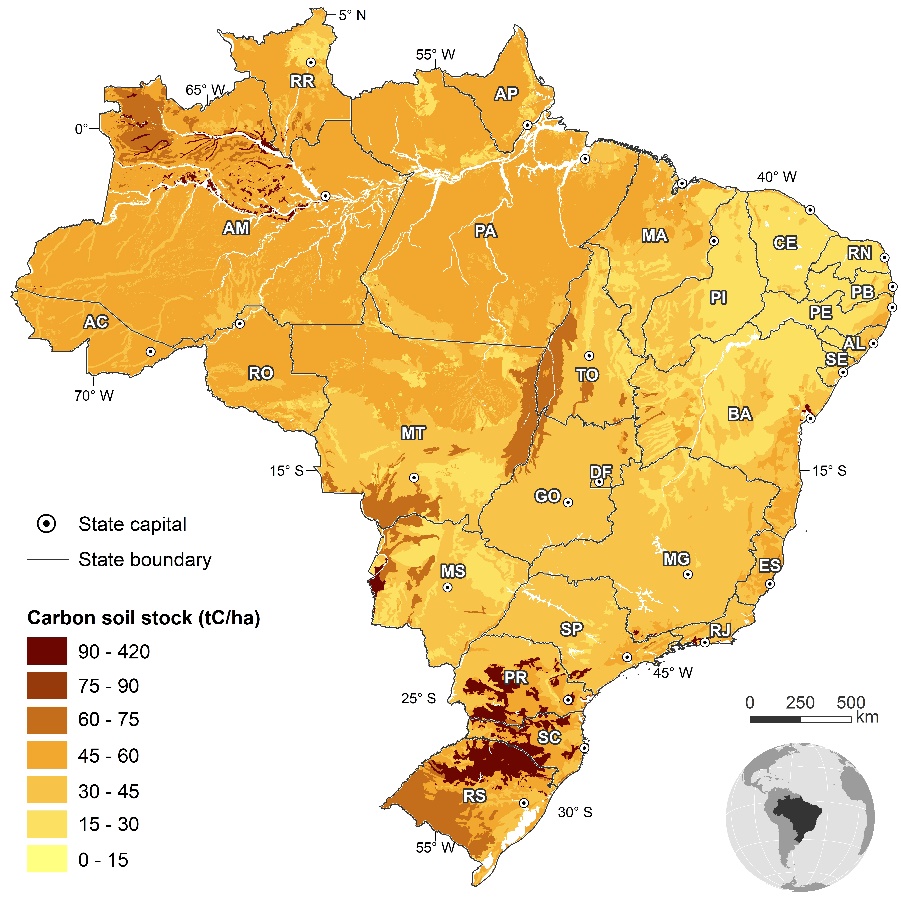
**

**Supplementary Figure S6:** Reference soil carbon stock (ton C/ha). Source: Bustamante et al.^8^. Map created using ArcGIS 10.8.1 (www.arcgis.com).

**Supplementary Table S8**: Carbon stocks (ton C/ha) by land use categories for Brazilian biomes. Source: Bustamante et al.^8^

| **Land use categories** | **Biome (ton C/ha)** | | | | | |
| --- | --- | --- | --- | --- | --- | --- |
|  | **Amazon** | **Cerrado** | **Atlantic Forest** | **Caatinga** | **Pampas** | **Pantanal** |
| Pasture | 7.57 | 7.57 | 7.57 | 4.09 | 6.35 | 7.57 |
| Annual agriculture | 5 | 5 | 5 | 5 | 5 | 5 |
| Perennial agriculture | 21 | 21 | 21 | 9 | 21 | 21 |
| Plantation forests (pine) | 87.03 | 87.03 | 87.03 | 87.03 | 87.03 | 87.03 |
| Plantation forests (eucalyptus) | 49.83 | 49.83 | 49.83 | 49.83 | 49.83 | 49.83 |
| Native vegetation in regeneration | 44% of original biomass | | | | | |

**Supplementary Table S9**: Carbon Removal factors (ton C/ha/year) by land use categories for Brazilian biomes. Source: Bustamante et al.^8^

| **Land use categories** | **Biome (ton C/ha)** | | | | | |
| --- | --- | --- | --- | --- | --- | --- |
|  | **Amazon** | **Cerrado** | **Atlantic Forest** | **Caatinga** | **Pampas** | **Pantanal** |
| Perennial crops growth | 2.6 | 2.6 | 2.6 | 1.8 | 2.6 | 2.6 |
| Grasslands in protected areas | 0.52 | 0.52 | 0.52 | 0.52 | 0.52 | 0.52 |
| Forests in protected areas | 0.43 | 0.2 | 0.32 | 0.1 | 0.2 | 0.32 |
| Forest regeneration (as forest as previous land use) | 4.96 | 1.72 | 5.35 | 0.6 | 1.76 | 2.77 |
| Forest regeneration (as Pastureland as previous land use) | 2.85 | 2.85 | 2.85 | 2.85 | 2.85 | 2.85 |
| Forest regeneration (as Cropland as previous land use) | 4.73 | 4.73 | 4.73 | 4.73 | 4.73 | 4.73 |
| Forest regeneration (as Other uses as previous land use) | 0.59 | 0.59 | 0.59 | 0.59 | 0.59 | 0.59 |

**Supplementary Table S10**: Soil carbon stock change factors (dimensionless) for each land use category. Source: Bustamante et al.^8^

| **Land use** | **Soil Carbon stock change factor for 20 years**  **(dimensionless)** |
| --- | --- |
|  |  |
| Native vegetation | 1 |
| Native vegetation in regeneration | 1 |
| Plantation forests | 0.673 |
| Pasture | 0.97 |
| Perennial agriculture | 1 |
| Full tillage agriculture | 0.612 |
| No tillage agriculture | 0.673 |
| Urban areas and water bodies | 0 |

**Eq. S1**: $\Delta C=\frac{\{C_{ref}*[{Fc}_{t0}-{Fc}_{t1}]\}}{D}$

Where:

ΔC is the annual change of carbon stock in the reservoir (ton C/yr);

*Cref* is the carbon stock in the soil of native vegetation (Mg C);

*Fct* is the stock change factor in the moment *t* (Table S11 - dimensionless); and

*D* is the time dependence of stock change factors which is the default time period for transition between equilibrium SOC values (years), here we use the IPCC default time of 20 years^11^.

## **S5.2 Details on fertilizers emission estimation**

To account for fertilizer emissions, it is considered a demand for nitrogen based on the recommendation of fertilization according to the level of productivity^12,13^. For the yield projections adopted, it is estimated utilization of 90 kg N/ha/year.

The equation for direct nitrous oxide emissions from the use of synthetic fertilizers is as follows (Eq. S2):

$$N_{2}O_{FERT i}=\left[ \left\{ N_{UREIA i}\times{(1-FRAC}_{\mathrm{GASFU}} \right) \right\}+\{\left( N_{FERT i}- N_{UREIA i} \right) \times{(1-FRAC}_{\mathrm{GASFU}})\}] \times\mathrm{EF}_{1} x 44/28$$

Where:

*N_2_O_FERT i_* is the nitrous oxide emission associated with the application of synthetic nitrogen fertilizers for year i (Kg - N_2_O / Kg of fertilizer applied)

*N_FERT i_ i*s the total amount of N applied as nitrogen fertilizer (kg) for year i

*N_UREIA i_* is the total amount of N applied as nitrogen fertilizer in the form of urea (kg) for year i

FRAC_GASFU_ is the fraction of N applied in the form of urea that volatilizes in the form of NH_3_ and NOx, being equal to 0.30

*FRAC_GASFO_* is the fraction of N applied in other sources that volatilizes in the form of NH_3_ and NOx (%), being equal to 0.10

*EF_1_* is the emission factor, being equal to 0.0100 kg N-N_2_O kg-1 N applied;

and *44/28* refers to the conversion from N to N_2_O

In addition, indirect N_2_O emissions from N added to soils as synthetic fertilizers are estimated. For this, emissions are considered due to the atmospheric deposition of N (NH_3_ and NOx) volatilized and deposited in soils and due to the N leachate and lost by surface runoff.

Indirect N_2_O emissions are calculated using the following formulas:

Atmospheric deposition of volatilized N (Eq. S3)

**N_2_O_D i_ = (N_FERT i_ x FRAC_GASF_ x EF_4_) x 44/18**

Where:

*N_2_O_D i_* is the nitrous oxide emission associated with atmospheric deposition from the application of synthetic nitrogen fertilizers (kg N_2_O / kg of fertilizer applied) for year i

*N_FERT_ i* is the total amount of N (in the form of urea or other fertilizers) applied as a nitrogen fertilizer (kg) for year i

*FRAC_GASF_* is the fraction of N applied (in the form of urea or other fertilizers) that volatilizes in the form of NH_3_ and NOx, being equal to 0.30 for urea and 0.10 for other fertilizers

*EF_4_* is the emission factor, being equal to 0.01 kg N-N_2_O / kg NH_3_-N and volatilized NOx-N

N leached and lost by runoff (Eq. S4)

**N_2_O_L i_ = (N_FERT i_ x FRAC_LEACH_ x EF_5_) x 44/18**

Where:

*N_2_O_L i_* is the nitrous oxide emission associated with leaching from the application of synthetic nitrogen fertilizers (kg N_2_O / kg of fertilizer applied) for year i

*N_FERT i_* is the total amount of N applied as a nitrogen fertilizer (kg) for year i

*FRAC_LEACH_* is the fraction of N lost by leaching and runoff, which is 0.30

*EF_5_* is the emission factor, being equal to 0.025 kg N-N_2_O / kg N leached or drained

## **S5.3. Details on limestone emission estimation**

To account for limestone emissions, an average limestone demand of 2 ton/ha is considered at the time of planting. For this, ages from 1 to 6 years are stipulated for sugarcane areas already established in 2018 according to Conab (2017)^14^, and it is considered that plantations are renewed, on average, every 6 years. Emissions are calculated as follows (Eq. S5):

$\mathbf{CO}_{\mathbf{2calc i}}\mathbf{=}\mathbf{Q}_{\mathbf{calc i}}\mathbf{*}\mathbf{FE}_{\mathbf{calc}}$ *** 44/12**

Where:

${CO}_{2calc i}$*i* = annual C emissions due to limestone application (ton of CO_2_) for year i

$Q_{calc i}$ = annual amount of limestone (calcitic + dolomitic) (ton) for year i

${FE}_{calc}$ = average emission factor for calcitic and dolomitic limestone, being equal to 0.125 10^3^ kg C for 10^3^ kg of limestone

*44/12* is the conversion factor from C to CO_2_

## **S5.4. Details on pre-harvest burning emission estimation**

To account for pre-harvest burning emissions, it was considered Conab (2020)^15^ information about harvest management per state. Emissions are calculated as follows (Eq. S6):

$$Lfire=A*MB*Cf*Gef*{10}^{-3}$$

Where:

*Lfire* = amount of CH_4_, CO, NOx and N_2_O gas emissions from burning waste, in tons (t)

*A* = burnt area, in hectares (ha)

*MB* = biomass available for combustion, in t / ha

*Cf* = combustion factor, dimensionless

*Gef* = emission factor, in g kg-1 of dry matter burnt. In which, available biomass (MB) is obtained using the equation described below:

*MB* = productivity (t / ha) * straw / stalk ratio

**Supplementary Table S11**: Parameters for estimating GHG emission from straw burning

|  | **Straw / stalk ratio** | **Combustion coefficient** | **Emission Factor**  **(kg of gas / kg of burnt biomass)** | | | |
| --- | --- | --- | --- | --- | --- | --- |
|  |  |  | **CH_4_** | **CO** | **N_2_O** | **NOx** |
| Parameter | 0.181 | 0.80 | 0.0027 | 0.092 | 0.00007 | 0.0025 |
| Source | PACKER et al. (2015)^16^ | IPCC (2006)^11^ | IPCC (2006)^11^ | | | |

**Supplementary references**

1. Philippidis, G. *et al*. The MAGNET Model Framework for Assessing Policy Coherence and SDGs: Application to the Bioeconomy; JRC Technical Reports, European Commission; EUR 29188 EN; Publications Office of the European Union: Luxembourg (2018).

2. Banse, M.; van Meijl, H.; Tabeau, A.; Woltjer, G. Will EU biofuel policies affect global agricultural markets? *Eur. Rev. Agric. Econ*. **35**, 117–141 (2008).

3. EurActiv. EU Parliament Ends Palm Oil and Caps Crop-Based Biofuels at 2017 Levels. (2018). Available online: https://www.euractiv.com/section/agriculture-food/news/eu-parliament-ends-palm-oil-and-caps-crop-based-biofuels-at-2017-levels/ (accessed on 16 March 2018).

4. Empresa de Pesquisa Energética (EPE), Ministério de Minas e Energia. Cenários de Oferta de Etanol e Demanda do Ciclo Otto 2018-2030. Brasília: EPE (2018).

5. Ministério da Agricultura Pecuária e Abastecimento (MAPA). Projeções do agronegócio: Brasil 2016/17 a 2026/27. Projeções de longo prazo. Brasília: MAPA (2017).

6. Instituto Brasileiro de Geografia e Estatística (IBGE). Produção Agrícola Municipal. Rio de Janeiro: IBGE (2020).

7. Rochedo, R. *et al*. The threat of political bargaining to climate mitigation in Brazil. *Nature Climate Change* **8**, 695–698 (2018).

8. Bustamante, M. *et al*. Emissões no setor uso da terra, mudança do uso da terra e florestas. In: Terceiro Inventário Brasileiro de Emissões e Remoções Antrópicas de Gases de Efeito Estufa. Brasília: MCTI. (2015).

9. SOS Mata Atlântica (SOSMA). Atlas da Mata Atlântica. São Paulo: SOSMA (2015).

10. Chave, J. *et al*. Error propagation and scaling for tropical forest biomass estimates. *Phil. Trans.: Biol. Sciences* **359**, 409-420, (2004).

11. The Intergovernmental Panel on Climate Change (IPCC). IPCC Guidelines for National Greenhouse Gas Inventories. National Greenhouse Gas Inventories Programme. Japan: IGES (2006).

12. Vitti, G., Mazza, J. Planejamento, estratégias de manejo e nutrição de cana-de-açúcar. *Informações Agronômicas* **97**, 1-16, (2002).

13. Rossetto, R., Dias, F. Nutrição e adubação da cana-de-açúcar: indagações e reflexões. *Informações Agronômicas* **110**, 6-11, (2005).

14. Companhia Nacional de Abastecimento (Conab). Perfil do Setor do Açúcar e do Etanol no Brasil. Edição para a safra 2014/15. Brasília: Conab (2017).

15. Companhia Nacional de Abastecimento (Conab). Acompanhamento da safra brasileira de cana-de-açúcar – v. 7 - Safra 2020/21, n. 2 - Segundo levantamento. Brasília: Conab (2020).

16. Packer, A., Vilela, V., Degaspari, I., Ramos, N. Queima de resíduos agrícolas. In: Terceiro Inventário Brasileiro de Emissões e Remoções Antrópicas de Gases de Efeito Estufa. Brasília: MCTI/Embrapa (2015).
